# Assessment of Left Ventricular Function in Cardiac MSCT Imaging by a 4D Hierarchical Surface-Volume Matching Process

**DOI:** 10.1155/IJBI/2006/37607

**Published:** 2006-06-25

**Authors:** Mireille Garreau, Antoine Simon, Dominique Boulmier, Jean-Louis Coatrieux, Hervé Le Breton

**Affiliations:** ^1^ Laboratoire Traitement du Signal et de l'Image, INSERM U642, Université de Rennes 1, Campus de Beaulieu, Rennes 35042, France; ^2^ Service d'Hémodynamique et de Cardiologie Interventionnelle, Centre Cardio-Pneumologique, CHU Pontchaillou, Rennes 35033, France

## Abstract

Multislice computed tomography (MSCT) scanners offer new perspectives for cardiac kinetics evaluation with 4D dynamic sequences of high contrast and spatiotemporal resolutions. A new method is proposed for cardiac motion extraction in multislice CT. Based on a 4D hierarchical surface-volume matching process, it provides the detection of the heart left cavities along the acquired sequence and the estimation of their 3D surface velocity fields. A Markov random field model is defined to find, according to topological descriptors, the best correspondences between a 3D mesh describing the left endocardium at one time and the 3D acquired volume at the following time. The global optimization of the correspondences is realized with a multiresolution process. Results obtained on simulated and real data show the capabilities to extract clinically relevant global and local motion parameters and highlight new perspectives in cardiac computed tomography imaging.


Hindawi Publishing Corporation
International Journal of Biomedical Imaging
Volume 2006, Article ID 37607, Pages 1–10
DOI 10.1155/IJBI/2006/37607

### Assessment of Left Ventricular Function in Cardiac MSCT Imaging by a 4D Hierarchical Surface-Volume Matching Process

**Mireille Garreau,[1] Antoine Simon,[1] Dominique Boulmier,[2] Jean-Louis Coatrieux,[1] and Hervé Le Breton[1,2]**

[1] *Laboratoire Traitement du Signal et de l'Image, INSERM U642, Université de Rennes 1, Campus de Beaulieu, 35042 Rennes, France*
[2] *Service d'Hémodynamique et de Cardiologie Interventionnelle, Centre Cardio-Pneumologique, CHU Pontchaillou, 35033 Rennes, France*

Received 1 December 2005; Revised 16 March 2006; Accepted 9 April 2006

Multislice computed tomography (MSCT) scanners offer new perspectives for cardiac kinetics evaluation with 4D dynamic sequences of high contrast and spatiotemporal resolutions. A new method is proposed for cardiac motion extraction in multislice CT. Based on a 4D hierarchical surface-volume matching process, it provides the detection of the heart left cavities along the acquired sequence and the estimation of their 3D surface velocity fields. A Markov random field model is defined to find, according to topological descriptors, the best correspondences between a 3D mesh describing the left endocardium at one time and the 3D acquired volume at the following time. The global optimization of the correspondences is realized with a multiresolution process. Results obtained on simulated and real data show the capabilities to extract clinically relevant global and local motion parameters and highlight new perspectives in cardiac computed tomography imaging.

Copyright © 2006 Mireille Garreau et al. This is an open access article distributed under the Creative Commons Attribution License, which permits unrestricted use, distribution, and reproduction in any medium, provided the original work is properly cited.

### 1. INTRODUCTION

Cardiovascular diseases cause the death of 17 million people every year, representing the major cause of mortality in industrialized countries. Various cardiac functional parameters are used to guide diagnosis, treatment, and followup of these diseases. The most used indicators (left ventricle volume (LVV), left ventricular mass (LVM), ejection fraction (EF)) provide information about the heart global function, but the detection and the treatment of some pathologies, such as atherosclerosis, would need the precise quantification of cardiac motion and deformation. Technological improvements in cardiac imaging provide rich opportunities for such a progress.

The minimally invasive assessment of heart motion has therefore been studied from modalities providing four-dimensional (4D) data sets. Magnetic resonance imaging (MRI), with cine MRI [1] and especially tagged MRI [2–4] and phase contrast MRI [5], has been extensively used, giving access to mid-wall deformations. However, its limited spatial resolution and long acquisition time prevent MRI to image both cardiac motion and coronary arteries. Ultrasound images [6, 7] providing, with high availability, cardiac sequences of high temporal resolution, are still limited by their low signal-to-noise ratio in spite of the recent advances of real-time 3D echocardiography. ECG-gated single photon emission computed tomography (SPECT) and positron emission tomography (PET) in spite of lower spatial and temporal resolutions have also been used in order to combine perfusion and contractility informations [8, 9]. The emergence of electron-beam computed tomography and of the dynamic spatial reconstructor (DSR) has provided opportunities for cardiac motion estimation [10–12], but their availability is still very limited. See [13] for an exhaustive review of cardiac image functional analysis methods.

The recent significant advances of multislice computed tomography (MSCT), with the introduction of ultra-fast rotating gantries (0.5 s/tr), multirows detectors, and retrospective ECG-gated reconstructions, provide high contrast and spatiotemporal resolutions and allow a huge progress towards the imaging of moving organs. These advances allow the observation of all cardiac structures simultaneously, for successive instants of the cardiac cycle, under one single breathhold. Some studies have been conducted in MSCT for the detection of coronary diseases [14, 15], but very few works have been realized for the quantitative 3D cardiac motion estimation [16].

The issue of nonrigid motion estimation from 3D images is one of the most important challenges of computer vision. Methods which have been proposed for this purpose can be classified into three kinds of approaches. In geometric model-based approaches, parametric models [3, 17, 18] involve the parametric formulation of the object and/or of the movement. This kind of methods is interesting to extract global motion and to represent it with few parameters. Nonparametric models [10, 19, 20], using mainly mass-spring and finite element methods, extract local motion using differential constraints. Optical flow methods [2, 11, 21, 22] are mostly based on intensity conservation and motion smoothing constraints. That constraint of intensity conservation with time is difficult to advance with MSCT data because of the contrast agent diffusion combined with the retrospective reconstruction of the sequence. Furthermore, these methods providing dense motion fields are difficult to handle with big data volumes in which the study deals with only few objects. Feature matching methods [1, 23, 24] are based on the search of correspondences between entities (considered at following times) according to descriptive parameters. These methods enable focusing the study on the objects of interest and extracting local motion. However, most of them are highly dependent from the segmentation quality because they need an accurate segmentation for each instant of the studied sequence.

We propose a new method to jointly extract ventricular shapes and their motion from cardiac MSCT images in one unique process. This problem of dual 3D shape and motion estimation is handled by a statistical approach provided by a Markov random field (MRF) [25], associated to a multiresolution process. The MRF theory has been extensively used in computer vision [26, 27]. Its application to motion analysis has mostly been done with optical flow estimation [28] and deformable models [29, 30].

In this paper, the 3D sparse nonrigid motion field to estimate at each time instant is formulated, in a Bayesian framework, as a Markov random field model under spatiotemporal regularity hypotheses. This motion field is provided by a matching method based on features of different types which are surface 3D mesh nodes at a first time instant and image voxels at the following time instant. These extracted motion fields can then be used for global and local motion quantification and interpretation. Results obtained on simulated and real data give satisfying results.

In the remainder of this paper, we describe in Section 2 the hierarchical surface-volume matching method we have developed including the preprocessing step, the definition of the Markov random field model and its application in a multiscale process, and the optimization stage. In Section 3 we present the results obtained on simulated and real data before concluding in Section 4.

### 2. A 4D HIERARCHICAL MOTION ESTIMATION METHOD

From a time sequence of 3D MSCT cardiac images, our approach allows the spatiotemporal detection of the left heart cavities and the quantification of their deformations. This is achieved by a multiscale matching method which provides, along the whole sequence, the correspondences between a 3D surface mesh extracted at one time instant and the 3D volume available at the next time instant. The overall method includes the following steps:

(a) a 3D segmentation step and a surface reconstruction process are first applied to only one 3D image of the time sequence (at time $t_0$) to provide the first surface of the sequence;
(b) a hierarchical surface-volume matching process is applied to estimate a 3D motion field between the surface at time $t_0$ and the next volume at time $t_1$;
(c) from the surface at time $t_0$ and the estimated motion field, a new 3D surface can be estimated at time $t_1$;
(d) steps (b) and (c) are repeated until all images of the sequence are processed.

In order to obtain the mesh corresponding to the first considered time of the sequence, step (a) is decomposed in this way: a segmentation tool, based on a 3D region growing process bounded by a gradient information, is applied [31]. The segmented surface is then reconstructed using the marching cubes algorithm. Finally, in order to prepare the matching process, the resulting surface mesh is regularized in such a manner that each node coordinates correspond to one volume voxel coordinates.

Surface estimation (step (c)) relies on the deformation of the surface corresponding to time $t_0$ with the motion estimated between times $t_0$ and $t_1$. A regularization step is then performed in order to fill mesh holes and to suppress redundant nodes.

The 3D motion field estimation (step (b)) is performed by a matching process applied between the surface representing the endocardium at time $t_0$ and the original volume corresponding to the next time $t_1$. A hierarchical process is used to gain both in terms of result quality and of computational efficiency. The matching process will firstly be described at one resolution, then the multiresolution scheme will be detailed.

### 2.1. A surface-volume matching process

In order to estimate the motion between one surface corresponding to time $t_0$ and a volume corresponding to the following time $t_1$, a surface-volume matching method has been developed.

A feature matching problem implies choosing the entities to match and to define local energies which can be combined to provide a distance measure between entities. As in almost all motion estimation issues, this measure is not sufficient to deal with a well-posed problem. It is therefore necessary to add contextual constraints. The best correspondences of selected entities can finally be obtained from the minimization of global energy.

One original contribution of this method is to establish correspondences between spatial entities which are not of the same nature: the matching process is conducted between 3D mesh nodes on the one hand and image voxels on the other hand.

The 3D motion field to compute between two successive instants is considered as a realization $f = \{f_i/i = 1, \ldots, N_S\}$ of a 3D random field $F$ ($N_S$ being the number of considered sites in the field). The set of sites $S$ of the field $F$ is given by all the 3D mesh nodes at time $t_0$. The labels assigned to these sites, expressed by the $f_i$ estimations, are given by the voxels found (at time $t_1$) in best correspondence with the 3D nodes.

According to Bayes' theory, the posterior probability of the realization $f$ according to the observation $d$ (the mesh nodes at time $t_0$ and the voxels at time $t_1$) is given by

$$p(f \mid d) = \frac{p(f)p(d \mid f)}{p(d)}, \tag{1}$$

with $p(f)$ the prior probability, $p(d \mid f)$ the conditional probability of the observation process $d$, and $p(d)$ the observation probability which is considered independent of $f$. According to the maximum a posteriori (MAP) estimator, the most probable realization $f$ is provided by the maximization of the a posteriori probability $p(f \mid d)$, with $p(f \mid d) \propto p(f)p(d \mid f)$.

The mechanical properties of the heart induce a spatiotemporal regularity of the motion field. To capture the spatial regularity of this motion, $p(f)$, used to model an a priori of the considered random field, is defined considering $F$ as a Markov random field.

This Markov random field (MRF) is defined in relation to a neighborhood system $\mu$. According to this definition, the neighborhood associated to one node $s$, noted $\mu_s$, is given by all nodes which share a common edge with the node $s$.

The MRF conditional probability models the local properties of the field according to this neighborhood. It is given by

$$p(f_s \mid f_{S-\{s\}}) = p(f_s \mid f_{\mu_s}). \tag{2}$$

From the neighborhood $\mu$, a set of cliques $C$ is defined as including all pairs of neighboring nodes:

$$C = \{\{s,t\} \in S^2,\ t \in \mu_s\}. \tag{3}$$

According to the Hammersley-Clifford theorem [32], the Markov random field $F$ in relation to the neighborhood system $\mu$ is also a Gibbs random field in relation to $\mu$. The prior probability distribution function is then given by

$$p(f) = \frac{1}{Z} \exp\{-U_R(f)\}, \tag{4}$$

with $Z$ a normalization constant and $U_R(f)$ a global energy function defining the interactions between the sites. $U_R(f)$ represents the internal energy of the random field and has a regularization effect. It is defined as

$$U_R(f) = \sum_{c \in C} V_R(c, f^c), \tag{5}$$

$V_R(c, f^c)$ being the local interaction potential defined for each clique $c$ and its associated labels $f^c = \{f_s,\ s \in c\}$. More precisely, we define $V_R$ by

$$V_R(c, f^c) = \alpha_R \frac{\|\vec{f_s} - \vec{f_t}\|}{\operatorname{dist}(s,t)} \quad \forall c = \{s,t\} \in C, \tag{6}$$

where $\alpha_R$ is a weighting factor, constant for every clique, $\vec{f_s}$ (resp., $\vec{f_t}$) is the motion vector estimated at site $s$ (resp., $t$), and $\operatorname{dist}(s,t)$ is the Euclidean distance between nodes $s$ and $t$.

The conditional probability density function $p(d \mid f)$ is given by the definition of a global data fidelity term which models the error between the realization $f$ and the observation $d$. It is defined by

$$p(d \mid f) = \exp\{-U_I(f,d)\}, \tag{7}$$

where $U_I(f,d)$ models the global estimation error. It is defined by

$$U_I(f,d) = \sum_{s \in S} V_I(s, f_s), \tag{8}$$

$V_I(s, f_s)$ being a local correspondence measure to evaluate the matching between one node $s$ at time $t_0$ ($s \in S$) and its corresponding voxel $f_s$ at time $t_1$. It provides a data conformity term and, in such a way, a distance between the observation $d$ (surface at time $t_0$ and 3D image at time $t_1$) and the estimated motion field given by $f$. For the analysis of correspondence between one node $s$ and one voxel $v$, this term is defined by the following equation:

$$V_I(s,v) = \alpha_d \cdot E_{\text{dist}}(s,v) + \alpha_c \cdot E_{\text{contour}}(v) + \alpha_t \cdot E_{\text{topol}}(s,v), \tag{9}$$

where

$$E_{\text{dist}}(s,v) = \operatorname{dist}(s,v),$$

$$E_{\text{contour}}(v) = C(v) = \begin{cases} 1 & \text{if } v \text{ belongs to a contour,} \\ 0 & \text{otherwise,} \end{cases} \tag{10}$$

$$E_{\text{topol}}(s,v) = \frac{1}{|\mu_s|} \sum_{i \in \mu_s} C\Big((v_x, v_y, v_z)^t + (s_x, s_y, s_z)^t - (i_x, i_y, i_z)^t\Big), \tag{11}$$

with $s$ is the considered node (of coordinates $(s_x, s_y, s_z)^t$); $v$ is the considered voxel (of coordinates $(v_x, v_y, v_z)^t$); $\operatorname{dist}(\cdot)$ is the Euclidean distance function; $C(\cdot)$ is the contour detection function (implemented by a Canny filtering); $\mu_s$ is the neighborhood of the node $s$; $i$ is a neighborhing node of $s$ (of coordinates $(i_x, i_y, i_z)^t$); and $\alpha_c$, $\alpha_t$, $\alpha_d$ are weighting factors.

From (1), (4), and (7), we have

$$p(f \mid d) \propto \exp\{-U(f,d)\}, \tag{12}$$

with

$$U(f,d) = \sum_{s \in S} V_I(s, f_s) + \sum_{c \in C} V_R(c, f_c) \tag{13}$$

a global energy which is to minimize to obtain the most probable correspondences.

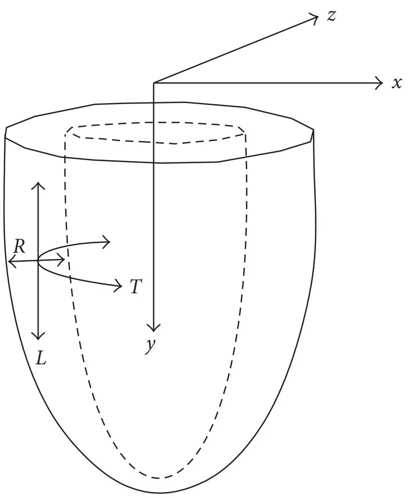

Figure 1: Cardiac motion components ($L$: longitudinal contraction, $R$: radial contraction, $T$: twisting).

### 2.2. Hierarchical scheme and optimization stage

The previously described motion extraction process is applied according to a hierarchical scheme which allows focusing the correspondence research area and reducing computing needs. This multiresolution scheme is considered to preserve local Markov property according to the high spatial resolution provided by MSCT data.

The surface mesh at time $t_0$ and the volume at next time $t_1$ are defined with decreasing scales as follows: each data set from upper resolution $R_i$ ($i = 1, \dots, n_l$, $n_l$ being the number of used resolution levels) is restricted in space at a lower level scale $R_{i-1}$ by the application of a mean filtering (or Gaussian filtering for the volume) and of a subsampling process in order to provide a regular mesh corresponding to volume voxel coordinates at the same level.

The matching process is first applied at the lowest resolution (with an initialization to a null motion) to guide the motion estimation with the coarsest details. The result of that first estimation is used as an initialization, after an interpolation step, for the correspondences computation at the next finer resolution. This motion extraction process is applied iteratively, with an adaptation of energy weighting coefficients, until the estimation is obtained at the desired resolution.

The global optimization of the correspondences is performed with a stochastic relaxation Metropolis algorithm combined with a simulated annealing process at the first lower-resolution level and with an iterated conditional mode (ICM) algorithm at the upper-resolution levels.

### 3. RESULTS

In a first part, the method has been applied on simulated data resulting from realistic deformations of a real ventricular shape. This first stage has provided the means to control and define the different parameters included in our approach. In a second part, the method has been applied on real human cardiac data acquired with MSCT. Finally, the means to extract global and local parameters in association to informative visual representation modes have been developed.

### 3.1. Tests on simulated data

Numerical simulations have been used to test the motion extraction process between two successive instants. In the absence of translation motion induced by patient respiration (the MSCT acquisition is realized under one single breath-hold) the heart is submitted to three main kinds of motion: radial and longitudinal contraction/expansion and twisting (cf. Figure 1). To simulate data, these three kinds of motion are applied to a previously 3D extracted mesh (corresponding to the first instant of the sequence). These deformations result in the mesh corresponding to the second instant. Then, this deformed mesh is inserted into a volume preprocessed by a Canny filter followed by an endocardial suppression step. The hierarchical matching process is finally applied between the surface before deformation and this volume at three increasing scales.

Using this simulation process, the real correspondences are known. It enables measuring the error of matching of the proposed method and to study the evolution of the matching process along iterations and at each stage of the hierarchical process. The impact of the different parameters involved in the computation of the energies or in the optimization process, as well as meaningful information provided by scale refinement, can also be evaluated.

Different tests have been conducted and combined to find the optimal value for each parameter involved in the matching process. These values have shown a good unicity (tests running with these values on data generated with varied motion parameters have provided optimal results) and a good stability (moderate variations of these values do not affect the quality of the result).

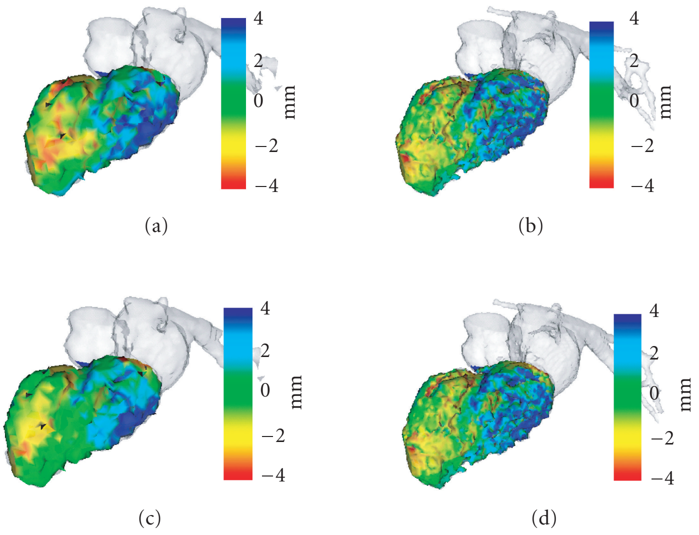

FIGURE 2: Simulated (a, b) and estimated (c, d) motion amplitudes with a left oblique anterior view at two resolutions (levels $64^3$ (a, c) and $128^3$ (b, d)) (colours: in blue (resp., red), motion directed outside (resp., inside) the cavity corresponding to positive (resp., negative) displacements), measures in millimeters.

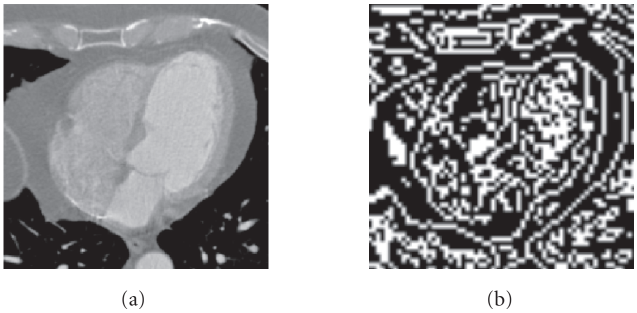

FIGURE 3: (a) One axial slice of one data volume of the sequence, (b) corresponding axial slice (at a lower resolution) after the Canny filtering process.

Figure 2 illustrates an example of results obtained at the two lowest-resolution levels ($R_1$ with volume size $64^3$ and $R_2$ ($128^3$)). In color are represented the applied (a, b) and estimated (c, d) motion amplitudes. In red are represented the displacements directed inside the cavity, and in blue the displacements directed outside the cavity. With an initial mean matching error of 6.7 mm at $R_1$ level, the process converges to a final mean error of 0.8 mm at $R_3$ level. We have observed that this hierarchical process enables gaining in precision and error deviation.

These results have been confirmed by tests running with different simulated motion parameters.

### 3.2. *Results on real data*

The algorithm has been applied on real human heart data with a temporal database acquired by a Siemens SOMATOM Sensation 16 with ten volume images representing a whole cardiac cycle. Each volume contains about 300 slices of $512 \times 512$ pixels, giving a resolution for each voxel of $0.35 \times 0.35 \times 0.5$ mm (cf. Figure 3(a) illustrating one CT axial slice).

The segmentation preprocess has been applied to the first volume of the sequence resulting in the extraction of the heart left cavities and of the beginning of the aorta. To obtain the surface mesh corresponding to time $t_0$, the segmented volume has been processed by the marching cubes algorithm (cf. Figure 4).

Using the optimal set of parameters found with the numerical simulations, the motion extraction process has been applied between this mesh (corresponding to time $t_0$) and the volume corresponding to time $t_1$ after a Canny operator application (cf. Figure 3(b) for an example of the Canny filter output). The algorithm has been iteratively applied to this dynamic sequence considering three resolution levels (from $64^3$ to $256^3$), providing a set of estimated surfaces and motion fields for each instant of the sequence.

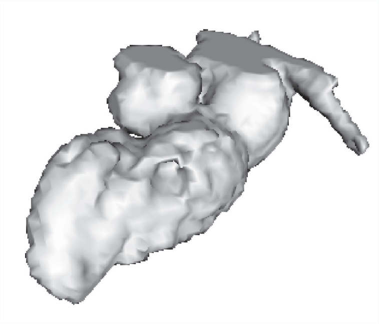

Figure 4: 3D surface of the extracted shape, corresponding to time $t_0$, represented at a lower resolution to highlight main structures.

Figure 5 illustrates results obtained at the two lowest levels at end-diastolic (a, b) and end-systolic (c, d) instants. The contraction movements (represented with red color or by negative displacements), characteristic of systole, are as well identified as the expansion movements (represented with blue color or by positive displacements) and are coherent with cardiac phases. We can see that the lowest level representation is meaningful and that the displacements obtained at upper levels provide the means to extract local clinical parameters at various scales.

Figure 6 shows the estimated motion amplitude during the whole cardiac cycle and using the highest resolution. We can observe that movements extracted on the whole sequence are coherent with cardiac phases. We can particularly remark the progressive expansion of the ventricle, from its basis to the apex, during the ventricle diastole (Figures 6(b)–6(f)), the important expansion occurring during the end-diastolic phase (Figure 6(f)), and the spatial evolution of contraction during the ventricle systole (Figures 6(g), 6(h)).

This kind of representations enables to highlight functional abnormalities. For instance, Figure 6 highlights the pathological situation where the antero-apical area (bottom left part of the visualized shape) suffers from akinesia.

### 3.3. Extraction of clinical parameters

Firstly, the extraction of global parameters of the ventricular function has been researched. After the interactive selection of three points determining the valvular plane, the 3D detected structure is limited to the left endocardium. Then, the ventricular volume can be computed for each instant, providing the curve of 3D volume variations along the entire temporal sequence analyzed. These measures give access to clinical functional features such as systolic volume or ejection fraction (EF). However, because the proposed method substracts trabeculas from the cavity volume, the measured volumes are lower than the volumes measured by a rough approximation of the endocardium. Obtained ejection fraction is therefore overestimated compared to those obtained by more classical methods (for one case, e.g., 55% was measured with echocardiography against 66% with our method).

Secondly, the extraction and representation of local parameters have been conducted. The anatomical system of reference, defined according to the great axis of the left ventricle, is computed from the patient specific 3D estimated surfaces. In this reference system, the detected movement is then decomposed in longitudinal, radial, and rotational motion components (cf. Figure 1). Cranial/caudal motion as well as inward and outward motion or wall twisting can therefore be measured. These motion components can then be analyzed along the temporal sequence, for a set of points selected on different anatomical parts of the ventricle (cf. Figures 7, 8). For this database, the extraction of radial movements show for example that the base of the heart verifies the largest motion amplitude (in contraction (negative values) and in expansion (positive values)) compared to the mid-cavity and to the apex. By the same way, movements of torsion can be locally enhanced and compared between different parts of the muscle. This kind of representation is of great interest for the clinical part because it allows to quantify how each type of movement (radial, longitudinal, torsion) is affected by specific pathologies.

A synthetic representation of local parameters has been also developed, based on the bull-eye representation, commonly used in echocardiography and MRI [33]. This kind of visualization is based on the anatomical segmentation of the 3D left ventricle in longitudinal and radial sectors and on their integration in a bull-eye scheme. The anatomical segmentation of the 3D surface is here conducted from the interactive selection of three points corresponding to the aortic and tricuspid valves and of the apex. For illustration, Figure 9 shows this anatomical segmentation and the motion amplitude estimated on the overall temporal sequence represented on the bull-eye scheme. This visual representation gives access to the dynamic behavior of each anatomical segment (on the whole sequence or between successive times). It allows to enhance and quantify pathological situations such as akinesia or asynchronism. The results observed on these real data show, for example, a reduced motion in apical septal segments (numbered 7, 8, 13, 14, and 17) which corresponds to the real pathological situation.

### 4. CONCLUSION

A new solution of motion extraction combined with surface estimation has been introduced and applied to the left ventricle in 4D cardiac MSCT imaging. It is based on a hierarchical surface-volume feature matching method formulated with a Markov random field and provides, with one unique process, the left cavity surfaces and associated 3D motion vector fields. The algorithm has been tested with simulated and real MSCT dynamic data, highlighting the great potential of MSCT imaging for quantitative clinical measure assessment in cardiac applications. Moreover these performances might be increased with new MSCT systems, combining more detectors (64 rows, or even 128 rows systems) and faster rotations. Indeed, with shorter acquisitions, the acquired data will be less submitted to artefacts, enabling to reconstruct more data volume by retrospective ECG-gating, providing

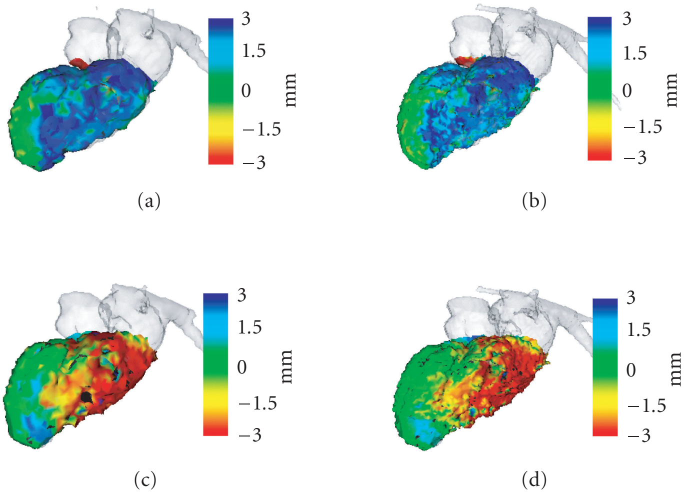

Figure 5: Estimated motion amplitude at end-diastolic (a, b) and end-systolic (c, d) instants, at two resolutions (levels $64^3$ (a, c) and $128^3$ (b, d)) (colours: in blue (resp., red), motion directed outside (resp., inside) the cavity corresponding to positive (resp., negative) displacements).

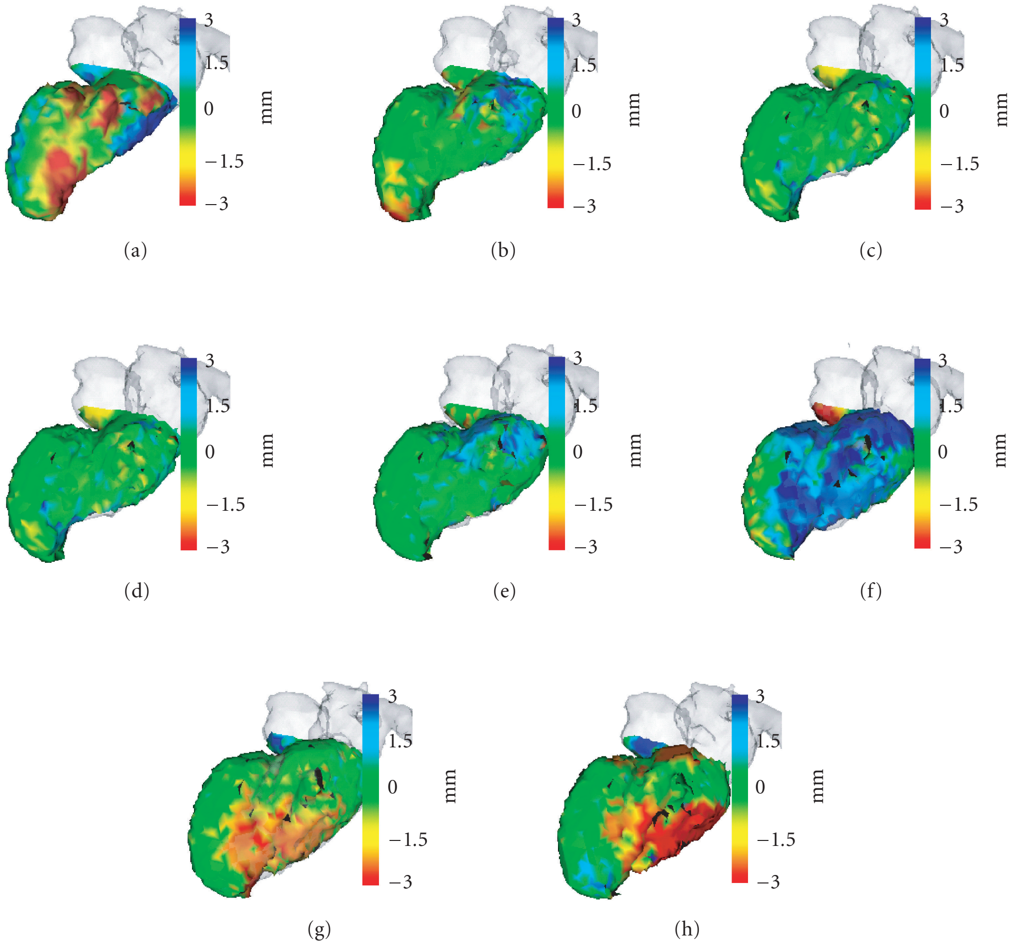

Figure 6: Estimated motion amplitude during the whole cardiac cycle. Time instants illustrated from (b)–(f) correspond to the ventricle diastole, while time instants from (g)–(a) correspond to the ventricle systole (colours: in blue (resp., red), motion directed outside (resp., inside) the cavity corresponding to positive (resp., negative) displacements).

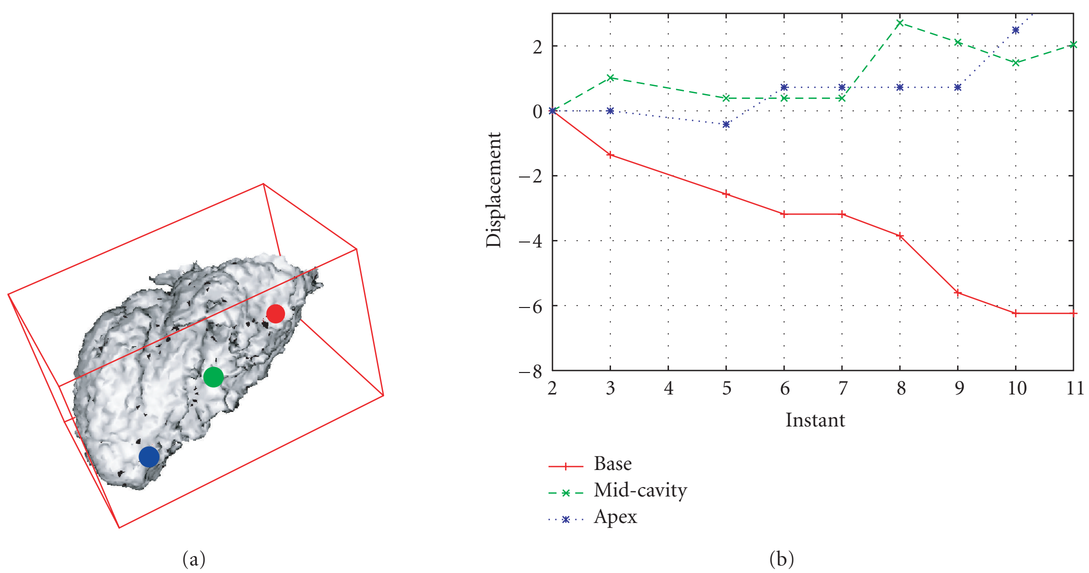

FIGURE 7: Longitudinal motion of three points interactively selected (base, mid-cavity, apex).

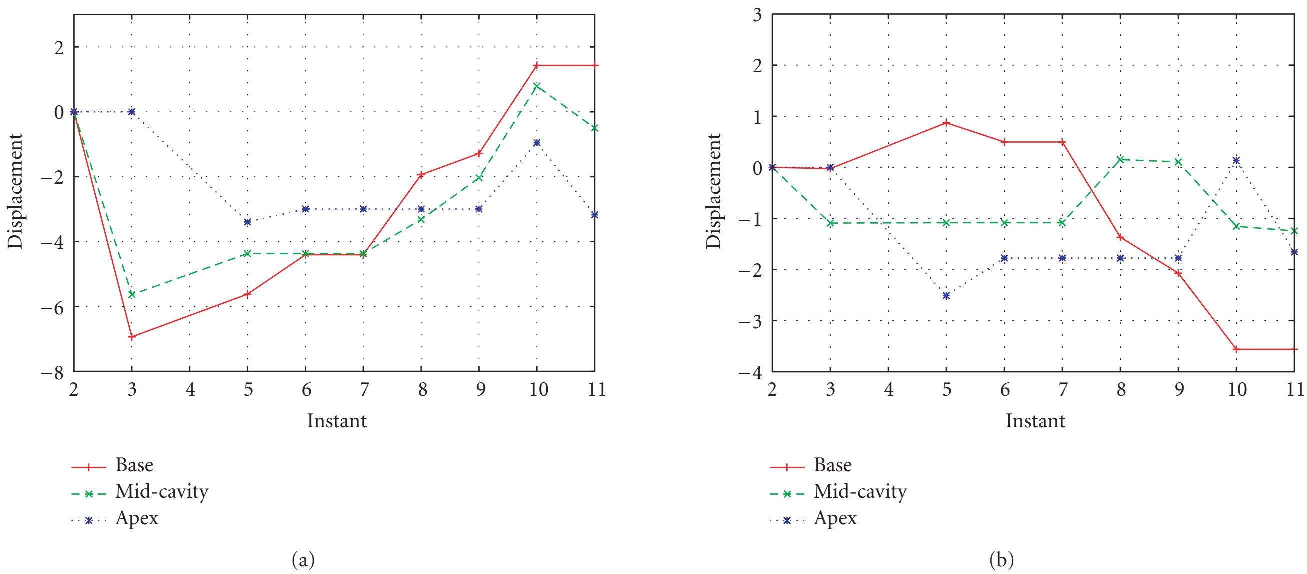

FIGURE 8: Radial and tangent motions of the three points represented in Figure 7.

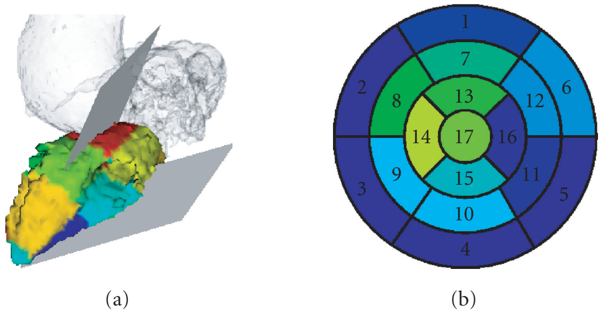

FIGURE 9: (a) Anatomical segmentation; (b) bull-eye representation of the estimated motion amplitude.

sequences of higher temporal resolution. Also, these new systems will be less sensitive to irregular heart rates. Further works will carry on extensive evaluation with normal and pathological real data, including especially a comparison with motion estimated with other imaging modalities.

### ACKNOWLEDGMENTS

This work is supported by Brittany region and the French Medical Research and Health National Institute (INSERM). The authors express their thanks to Siemens Medical Division, France, for having provided us real MSCT image data.

### REFERENCES

[1] P. Shi, A. J. Sinusas, R. T. Constable, E. T. Ritman, and J. S. Duncan, "Point-tracked quantitative analysis of left ventricular surface motion from 3-D image sequences," *IEEE Transactions on Medical Imaging*, vol. 19, no. 1, pp. 36–50, 2000.

[2] J. L. Prince and E. R. McVeigh, "Motion estimation from tagged MR image sequences," *IEEE Transactions on Medical Imaging*, vol. 11, no. 2, pp. 238–249, 1992.

[3] J. Park, D. Metaxas, and L. Axel, "Analysis of left ventricular wall motion based on volumetric deformable models and MRI-SPAMM," *Medical Image Analysis*, vol. 1, no. 1, pp. 53–71, 1996.

[4] P. Clarysse, M. Han, P. Croisille, and I. E. Magnin, "Exploratory analysis of the spatio-temporal deformation of the myocardium during systole from tagged MRI," *IEEE Transactions on Biomedical Engineering*, vol. 49, no. 11, pp. 1328–1339, 2002.

[5] F. G. Meyer, R. T. Constable, A. J. Sinusas, and J. S. Duncan, "Tracking myocardial deformation using phase contrast MR velocity fields: a stochastic approach," *IEEE Transactions on Medical Imaging*, vol. 15, no. 4, pp. 453–465, 1996.

[6] X. Papademetris, A. J. Sinusas, D. P. Dione, and J. S. Duncan, "Estimation of 3D left ventricular deformation from echocardiography," *Medical Image Analysis*, vol. 5, no. 1, pp. 17–28, 2001.

[7] W. Yu, N. Lin, P. Yan, et al., "Motion analysis of 3D ultrasound texture patterns," in *Proceedings of 2nd International Workshop on Functional Imaging and Modeling of the Heart (FIMH '03)*, pp. 252–261, Lyon, France, June 2003.

[8] G. Germano, J. Erel, H. Lewin, P. B. Kavanagh, and D. S. Berman, "Automatic quantitation of regional myocardial wall motion and thickening from gated technetium-99m sestamibi myocardial perfusion single-photon emission computed tomography," *Journal of the American College of Cardiology*, vol. 30, no. 5, pp. 1360–1367, 1997.

[9] T. R. Miller, J. W. Wallis, B. R. Landy, R. J. Gropler, and C. L. Sabharwal, "Measurement of global and regional left ventricular function by cardiac PET," *Journal of Nuclear Medicine*, vol. 35, no. 6, pp. 999–1005, 1994.

[10] S. Benayoun and N. Ayache, "Dense non-rigid motion estimation in sequences of medical images using differential constraints," *International Journal of Computer Vision*, vol. 26, no. 1, pp. 25–40, 1998.

[11] J.-M. Gorce, D. Friboulet, and I. E. Magnin, "Estimation of three-dimensional cardiac velocity fields: assessment of a differential method and application to three-dimensional CT data," *Medical Image Analysis*, vol. 1, no. 3, pp. 245–261, 1997.

[12] C. D. Eusemann, E. L. Ritman, and R. A. Robb, "Parametric visualization methods for the quantitative assessment of myocardial motion," *Academic Radiology*, vol. 10, no. 1, pp. 66–76, 2003.

[13] A. F. Frangi, W. J. Niessen, and M. A. Viergever, "Three-dimensional modeling for functional analysis of cardiac images: a review," *IEEE Transactions on Medical Imaging*, vol. 20, no. 1, pp. 2–25, 2001.

[14] S. Schroeder, A. F. Kopp, B. Ohnesorge, et al., "Accuracy and reliability of quantitative measurements in coronary arteries by multi-slice computed tomography: experimental and initial clinical results," *Clinical Radiology*, vol. 56, no. 6, pp. 466–474, 2001.

[15] A. Larralde, C. Boldak, M. Garreau, C. Toumoulin, D. Boulmier, and Y. Rolland, "Evaluation of a 3D segmentation software for the coronary characterization in multi-slice computed tomography," in *Proceedings of 2nd International Workshop on Functional Imaging and Modeling of the Heart (FIMH '03)*, vol. 2674 of *Lecture Notes in Computer Science*, pp. 39–51, Lyon, France, June 2003.

[16] M. Garreau, A. Simon, D. Boulmier, and H. Guillaume, "Cardiac motion extraction in multislice computed tomography by using a 3D hierarchical surface matching process," in *Proceedings of IEEE Computers in Cardiology (CinC '04)*, pp. 549–552, Chicago, Ill, USA, September 2004.

[17] C. W. Chen, T. S. Huang, and M. Arrott, "Modeling, analysis, and visualization of left ventricle shape and motion by hierarchical decomposition," *IEEE Transactions on Pattern Analysis and Machine Intelligence*, vol. 16, no. 4, pp. 342–356, 1994.

[18] E. Bardinet, L. D. Cohen, and N. Ayache, "A parametric deformable model to fit unstructured 3D data," *Computer Vision and Image Understanding*, vol. 71, no. 1, pp. 39–54, 1998.

[19] W.-C. Huang and D. B. Goldgof, "Adaptive-size meshes for rigid and nonrigid shape analysis and synthesis," *IEEE Transactions on Pattern Analysis and Machine Intelligence*, vol. 15, no. 6, pp. 611–616, 1993.

[20] I. Cohen, N. Ayache, and P. Sulger, "Tracking points on deformable objects using curvature information," in *Proceedings of the 2nd European Conference on Computer Vision (ECCV '92)*, pp. 458–466, Santa Margherita Ligure, Italy, May 1992.

[21] S. M. Song and R. M. Leahy, "Computation of 3-D velocity fields from 3-D cine CT images of a human heart," *IEEE Transactions on Medical Imaging*, vol. 10, no. 3, pp. 295–306, 1991.

[22] P. Hellier, C. Barillot, E. Mémin, and P. Pérez, "Hierarchical estimation of a dense deformation field for 3-D robust registration," *IEEE Transactions on Medical Imaging*, vol. 20, no. 5, pp. 388–402, 2001.

[23] A. A. Amini and J. S. Duncan, "Bending and stretching models for LV wall motion analysis from curves and surfaces," *Image and Vision Computing*, vol. 10, no. 6, pp. 418–430, 1992.

[24] C. Kambhamettu, D. B. Goldgof, M. He, and P. Laskov, "3D nonrigid motion analysis under small deformations," *Image and Vision Computing*, vol. 21, no. 3, pp. 229–245, 2003.

[25] A. Simon, M. Garreau, D. Boulmier, J.-L. Coatrieux, and H. Le Breton, "A surface-volume matching process using a Markov random field model for cardiac motion extraction in MSCT imaging," in *Proceedings of 3rd International Workshop on Functional Imaging and Modeling of the Heart (FIMH '05)*, pp. 457–466, Barcelona, Spain, June 2005.

[26] S. Z. Li, *Markov Random Field Modeling in Computer Vision*, Springer Computer Science Workbench Series, Springer, London, UK, 1995.

[27] P. Pérez, "Markov random fields and images," *CWI Quarterly*, vol. 11, no. 4, pp. 413–437, 1998.

[28] R. Fablet and P. Bouthemy, "Motion recognition using non-parametric image motion models estimated from temporal and multiscale co-occurrence statistics," *IEEE Transactions on Pattern Analysis and Machine Intelligence*, vol. 25, no. 12, pp. 1619–1624, 2003.

[29] C. Kervrann and F. Heitz, "Statistical deformable model-based segmentation of image motion," *IEEE Transactions on Image Processing*, vol. 8, no. 4, pp. 583–588, 1999.

[30] M. Mignotte, J. Meunier, and J.-C. Tardif, "Endocardial boundary estimation and tracking in echocardiographic images using deformable templates and Markov random fields," *Pattern Analysis & Applications*, vol. 4, no. 4, pp. 256–271, 2001.

[31] H. Guillaume and M. Garreau, "Segmentation de cavités cardiaques en imagerie scanner multi-barettes," in *Forum des Jeunes Chercheurs en Génie Biologique et Médical*, pp. 92–93, Nantes, France, May 2003.

[32] J. Besag, "Spatial interaction and the statistical analysis of lattice systems," *Journal of the Royal Statistical Society. Series B*, vol. 36, no. 2, pp. 192–236, 1974.

[33] M. D. Cerqueira, N. J. Weissman, V. Dilsizian, et al., "Standardized myocardial segmentation and nomenclature for tomographic imaging of the heart," *Circulation*, vol. 105, no. 4, pp. 539–542, 2002.

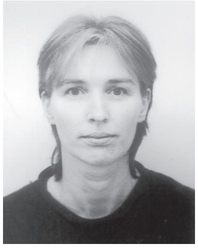

**Mireille Garreau** was born in 1962. She received her M.S. degree in signal processing and telecommunications from the University of Rennes 1 in 1985. She obtained a Ph.D. degree in signal and image processing and telecommunications from the University of Rennes1 in 1988. She has been working as an Associate Professor since 1989 and as a Professor since 2004. She has been involved in biomedical engineering for about 15 years. Her main research topics are related to the joint use of biomedical knowledge engineering (or artificial intelligence in medicine) and image analysis (generic methods going from calibration to segmentation, from 3D reconstruction to motion estimation and image understanding) with applications to vascular and cardiac imaging. She is the Head of the ACTIVE project (tissue analysis and characterization in vascular imaging) in the LTSI (joint research laboratory of INSERM and University of Rennes1, France).

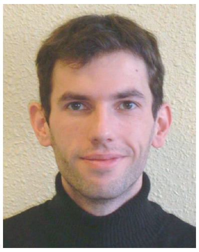

**Antoine Simon** was born in 1978 in Brest, France. He graduated from the Ecole Suprieure de l'Ouest (ESEO) in 2002 and received the M.S. degree in image and signal processing in biology and medicine from the Faculty of Medicine, Angers. He then joined the Laboratory of Signal and Image Processing (LTSI, U642 INSERM, University of Rennes1, France) where he obtained a Ph.D. degree in 2005. His main research interests are related to cardiac imaging, motion extraction, characterization and representation, and multiresolution image processing.

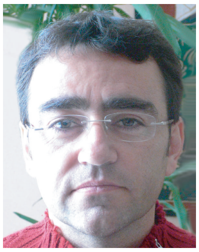

**Dominique Boulmier** was born in 1968. He joined the Rennes University of Medicine between 1986 and 1994. He was a Fellow in cardiology between 1994 and 2000 and an Assistant Professor between 2000 and 2003. He has been working since then as an interventional cardiologist at the Rennes University Hospital (Bretagne). He is involved in clinical research on cardiac imaging with computed tomography.

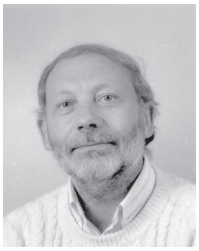

**Jean-Louis Coatrieux** received the degree in electrical engineering from the Polytechnic Institute, Grenoble, France, in 1970 and the Ph.D. and State Doctorate degrees in sciences in 1973 and 1983, respectively, from the University of Rennes 1, France. He was an Assistant Professor from 1970 to 1975 and an Associate Professor from 1976 to 1986 at the Institute of Technology of Rennes. Since 1986, he has been the Director of Research at the National Institute for Health and Medical Research (INSERM), France, and since 1993 has been a Professor at the New Jersey Institute of Technology, Newark. He has been the Head of the Laboratoire Traitement du Signal et de l'Image, INSERM, up to 2003 and in charge of the National Research Program in Health Technology at the Ministry of Research (1998–2001), France. He was President of the joint Committee CNRS-INSERM in this area (2001–2003). His experience is related to 3D images, signal processing, computational modeling, and complex systems with applications in integrative biomedicine. He has served as the Editor-in-Chief of the IEEE Transactions on Biomedical Engineering (1996–2000). He has received several awards from IEEE (EMBS Service Award, 1999, and the Third Millennium Award, 2000) and he is Doctor Honoris Causa from the University of Nanjing, China.

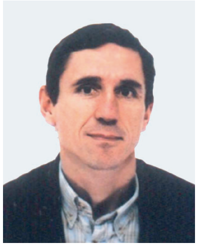

**Hervé Le Breton** was born in 1959. He went to the Rouen University of Medicine between 1978 and 1984. He was a Fellow in cardiology from 1984 to 1988 and has been working as an interventional cardiologist from 1989 at the Rennes University Hospital (Bretagne). He spent one year at the Cleveland Clinic Foundation in 1994-1995 doing animal research (role of antiplatelet agents in restenosis post PTCA). He was nominated Professor of Medicine in 1999. His main research concerns are in coronary artery disease and the field of interventional cardiology.

